# Minimally invasive stabilization of the anterior pelvic ring in fragility fractures using a submuscularly implanted internal fixator – a retrospective case series of 34 geriatric patients

**DOI:** 10.1007/s00068-025-02893-9

**Published:** 2025-06-16

**Authors:** Julia Lenz, Carmie Schneider, Ludwig Oberkircher, Vanessa Ketter, Tom Knauf, Steffen Ruchholtz, Juliana Hack

**Affiliations:** 1 Marburg University, School of Medicine, Center for Orthopaedics and Trauma Surgery, University Hospital Gießen and Marburg GmbH, Baldingerstraße, 35043 Marburg, Germany; 2 Orthopaedic and Trauma Surgery, Lahn-Dill Clinics, Wetzlar, Germany; 3 Department for Trauma Surgery, Orthopaedic Surgery and Arthroplasty, Medizin Campus Bodensee, Friedrichshafen, Germany; 4 Orthopaedics and Trauma Surgery, Helios Hospital Kassel, Kassel, Germany

**Keywords:** Anterior pelvic ring fractures, Fragility fractures of the pelvis, Internal fixation, Minimally invasive technique, Geriatric patients

## Abstract

**Purpose:**

Various surgical techniques for osteosynthesis in fragility fractures of the pelvis (FFP) are described. Since 2012, a submuscularly placed internal fixator has been used to stabilize the anterior pelvic ring. Indications for this procedure are a dislocation in the anterior pelvic ring and/or severe pain with associated immobility. This technique potentially has several advantages compared to subcutaneous procedures, including improved patient comfort, less irritation, and enhanced biomechanical stability due to the deeper rod placement.

**Methods:**

Digital files of patients aged ≥ 65 years, who were treated with an internal fixator at a Level I trauma center in Germany between 2012 and 2021, were retrospectively analyzed.

**Results:**

Thirty-four patients (median age 79 years, 77% female, 61.8% ASA III) were treated. Most fractures were caused by ground-level falls (64.7%), followed by road traffic accidents (11.8%). In patients with low impact trauma, the most common fracture types were FFP IIb (37.04%) and FFP IIIc (18.52%). Complications during surgery occurred in 4 patients (11.76%) and postoperative complications in 6 patients (17.6%), with hematoseroma being the most common. Non-surgical complications occurred in 20 patients (total: 58.8%; Clavien-Dindo type 2 in 70%). After 12 months, the majority of all patients had the same mobility level as before the fracture.

**Conclusion:**

The submuscularly placed internal fixator is an effective technique for stabilizing anterior pelvic ring fractures in geriatric patients, offering advantages in cases of high dorsoventral instability or persistent severe anterior pain.

## Introduction

Different epidemiological studies have shown an age older than 65 years in 25% of the world’s population by the year 2030 [1, 2]. Because of the constancy in demographic changes, the incidence of fractures in the elderly is still rising.

As one of the most common geriatric fractures, the fragility fracture of the pelvis (FFP) with its increasing incidence is a daily clinical challenge [1, 3]. While evidence for the treatment of hip fractures as the “leader” of geriatric fractures already exists [4], there are just a very small number of prospective studies and long-term results concerning the therapy of fragility fractures of the pelvis.

In contrast to pelvic fractures of younger adults as a result of high- energy trauma, fragility fractures of geriatric patients are usually caused by a ground-level fall and often associated with the presence of osteoporosis [5–9].

Osteoporosis leads to a considerable decrease in bone mineral density [10]. As a result, the pelvic bone is less resistant to external and internal forces, with an increased risk for both suffering fractures and the failure of osteosynthesis [11–13]. In addition, demographic changes are leading to higher activity levels in older people, resulting in a higher number of high impact traumas in the geriatric population.

Addressing these considerations of pelvic fractures in the geriatric population, the FFP *(fragility fracture of the pelvis)* classification was established to provide recommendations for the best treatment [7, 14]. While a conservative therapy, including physiotherapy and an adequate pain therapy, should be focused upon stable fractures, surgical stabilization is recommended in unstable and dislocated fractures or if the conservative treatment has failed [7, 14].

Therefore, various surgical techniques have been developed in the past, with a focus on minimally invasive procedures, in order to minimize perioperative complications [15, 16].

For minimally invasive stabilization of the anterior pelvic ring in the presence of dislocation and instability, the external fixator is still considered to be the gold standard procedure, especially because of its ease of use, the short duration of surgery and the global availability [17]. But this well-known technique has its disadvantages, such as a high risk of pin tract infections; patient discomfort; and its negative influence on an early mobilization [18–20], which should be the main goal of the treatment, especially in geriatric patients [15, 16, 21]. So there is still no clear recommendation to use external fixation in geriatric patients [14].

Due to this, newer techniques for a minimally invasive subcutaneous internal stabilization of the anterior pelvic ring have been published in the past [22–25]. As a common complication of this technique, the authors mention the irritation of the lateral femoral nerve in up to 57% of cases [22], so a second surgery for implant removal is often necessary. Another minimally invasive technique, using retrograde transpubic screws for pubic ramus fractures, shows favorable results but only in cases of minimally displaced fractures [25].

Taking this into consideration, a minimally invasive technique was established to stabilize dislocated fractures of the anterior pelvic ring using an internal fixator, which consists of a submuscularly placed curved titanium rod fixed with bilaterally positioned supraacetabular polyaxial pedicle screws. This technique offers all of the advantages of minimally invasive procedures and is a definitive stabilization option. With its placement underneath the abdominal muscles, it potentially offers increased comfort and less irritations of the superficial nerves. Moreover, in a biomechanical study, a high level of biomechanical stability was shown even in the osteoporotic pelvis by placing the titanium rod close to the bone [26].

The purpose of the present study was to analyze all patients treated in our hospital with this new surgical technique regarding early intra- and postoperative complications and clinical outcome.

## Materials and methods

### Patients and study design

The medical records of 34 geriatric patients (age ≥ 65 years) treated with the submuscular internal fixator between 2012 and 2021 at a Level I trauma center in Germany were retrospectively reviewed. No patients were excluded, and 33 patients underwent a follow-up examination after a minimum of 6 weeks. One patient was lost to follow-up due to death during hospital stay. Approval was obtained from the local ethics committee (AZ RS 22/21).

## Indication and contraindication

The indications for this stabilization technique were on the one hand a pelvic ring fracture with severe pain in the anterior ring, which leads to an increasing immobility, and on the other hand an unstable fracture with a ventral dislocation, in which the surgeon believed there was a need for anterior fixation. A ventral dislocation was defined as a displacement of the pubic bone by at least the width of the shaft in cases of unilateral or bilateral fractures, in combination with pain in the affected area.

The decision regarding the most appropriate treatment was based on an in-house treatment algorithm for patients with FFP at the author’s institution. Conservative treatment was considered a failure with a VAS of 5 or more, despite adequate analgesics [16]. Hemodynamic instability was a contraindication for this procedure.

## Operative technique and postoperative protocol

**Fig. 1:**
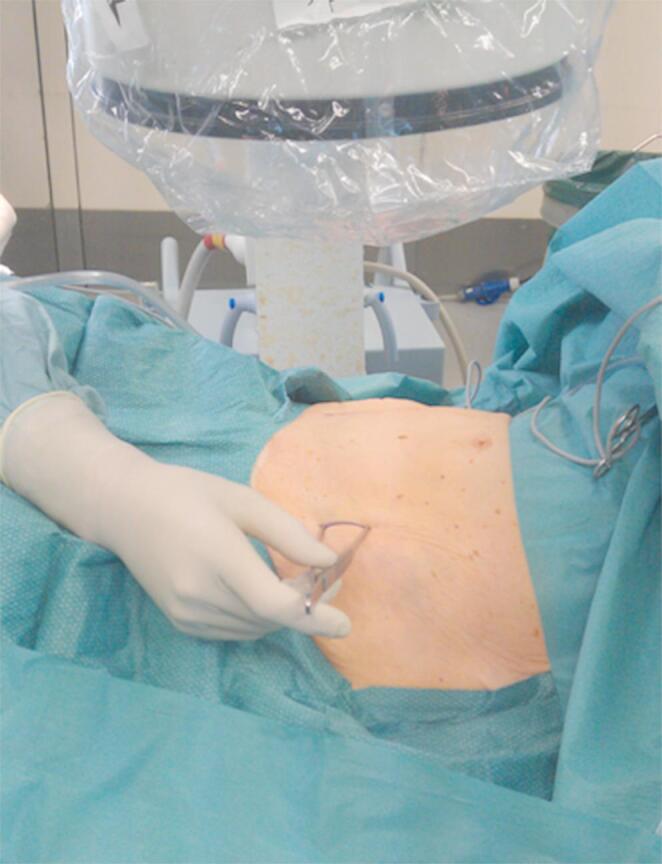
The correct incision point is identified using fluoroscopy

**Fig. 2:**
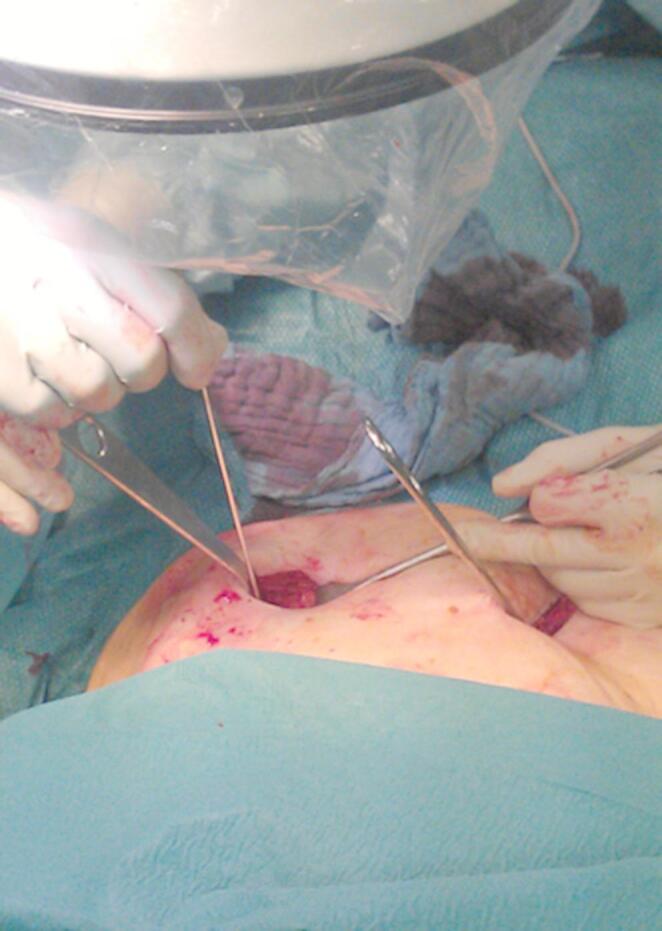
The external iliac vessels are hold to the medial side and the psoas muscle, the iliacus muscle and the femoral nerve are hold to the lateral side using blunt retractors

**Fig. 3:**
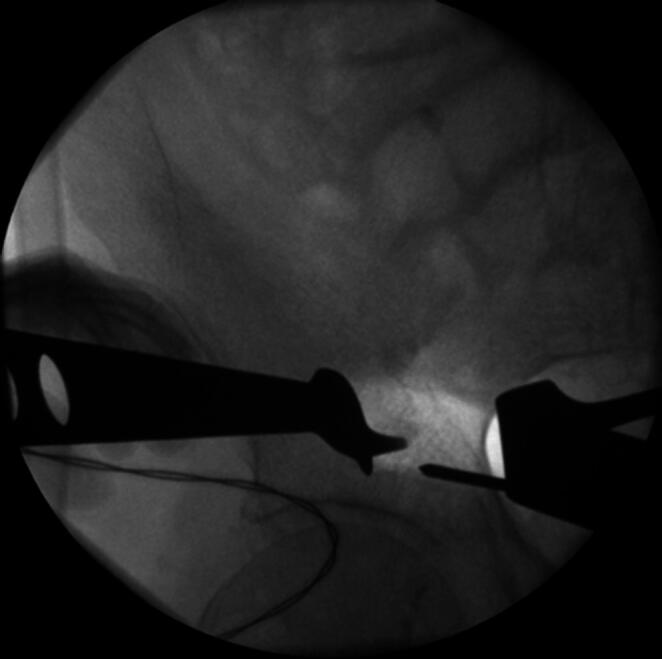
The screw-entry point is identified using fluoroscopy

**Fig. 4:**
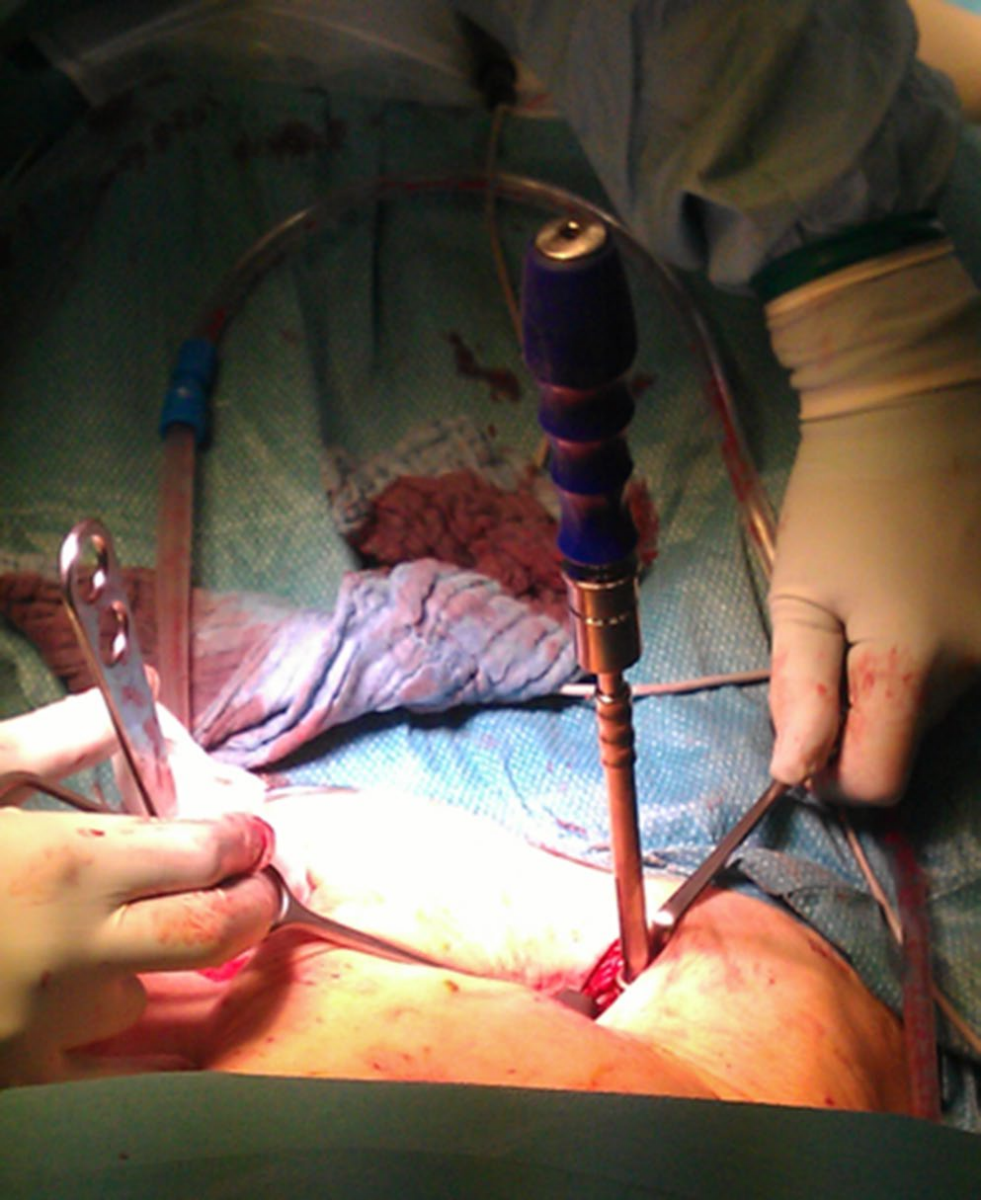
A polyaxial screw is inserted to the supra-acetabular region

The patient is positioned supine on a flat radiolucent table, and general anesthesia is administered. In order to neutralize the iliopsoas muscle and to relax the femoral nerve, both hips are bent at an angle of 30 to 40 degrees. The patient’s pelvis is widely sterilely prepared and draped. A C-arm is used to identify the correct incision point for the minimally- invasive transabdominal, retroperitoneal approach (Fig. 1). A longitudinal 3-cm mini- incision is made in the proximal one third of the linea arcuata of the iliac bone. Thereafter a blunt dissection of the abdominal muscles is performed. Blunt retractors are inserted that hold the external iliac vessels to the medial side and the psoas muscle, the iliacus muscle and the femoral nerve to the lateral side (Fig. 2). Now there is a direct view to the supra-acetabular region to insert a polyaxial screw of 7.5 mm diameter and usually 50 mm to 55 mm length *(Marquardt Medizintechnik GmbH*,* Spaichingen*,* Germany)*(Figs. 3 and 4). Screw position is confirmed with fluoroscopy. The same procedure is repeated for the contralateral side.

**Fig. 5:**
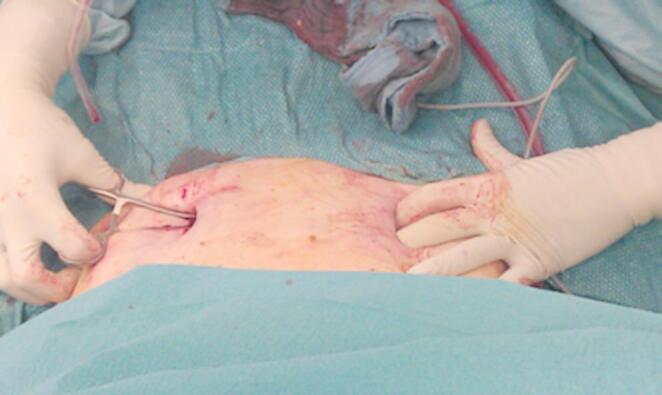
To prepare the insertion of the titanium rod, blunt preparation of the soft tissues is performed using both superior pubic rami as landmarks for the preparation while the external iliac vessels are mobilized anteriorly

**Fig. 6:**
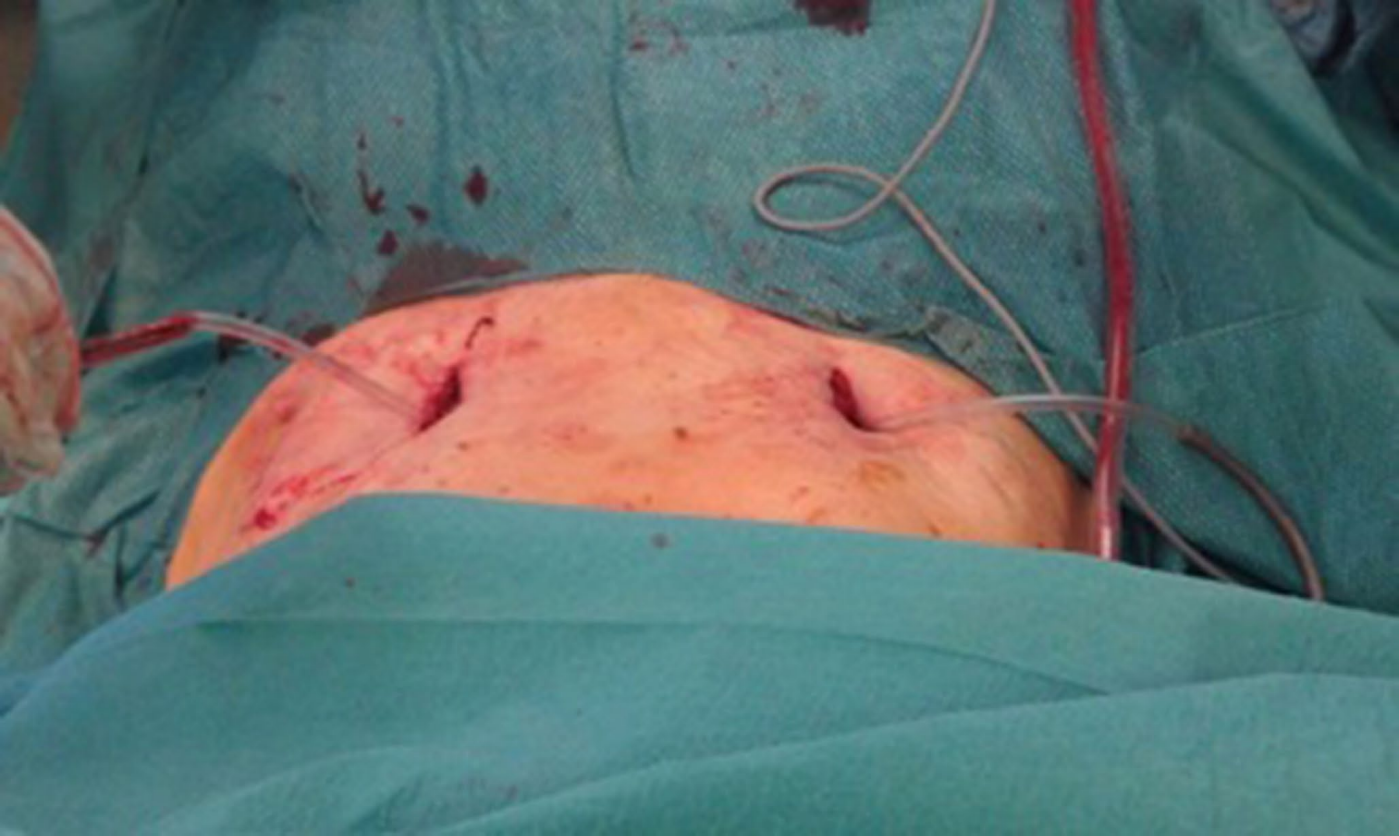
A soft drainage is inserted on one side and tunneled through the soft tissues along the superior pubic rami posteriorly to the external iliac vessels until it reaches the contralateral incision

**Fig. 7:**
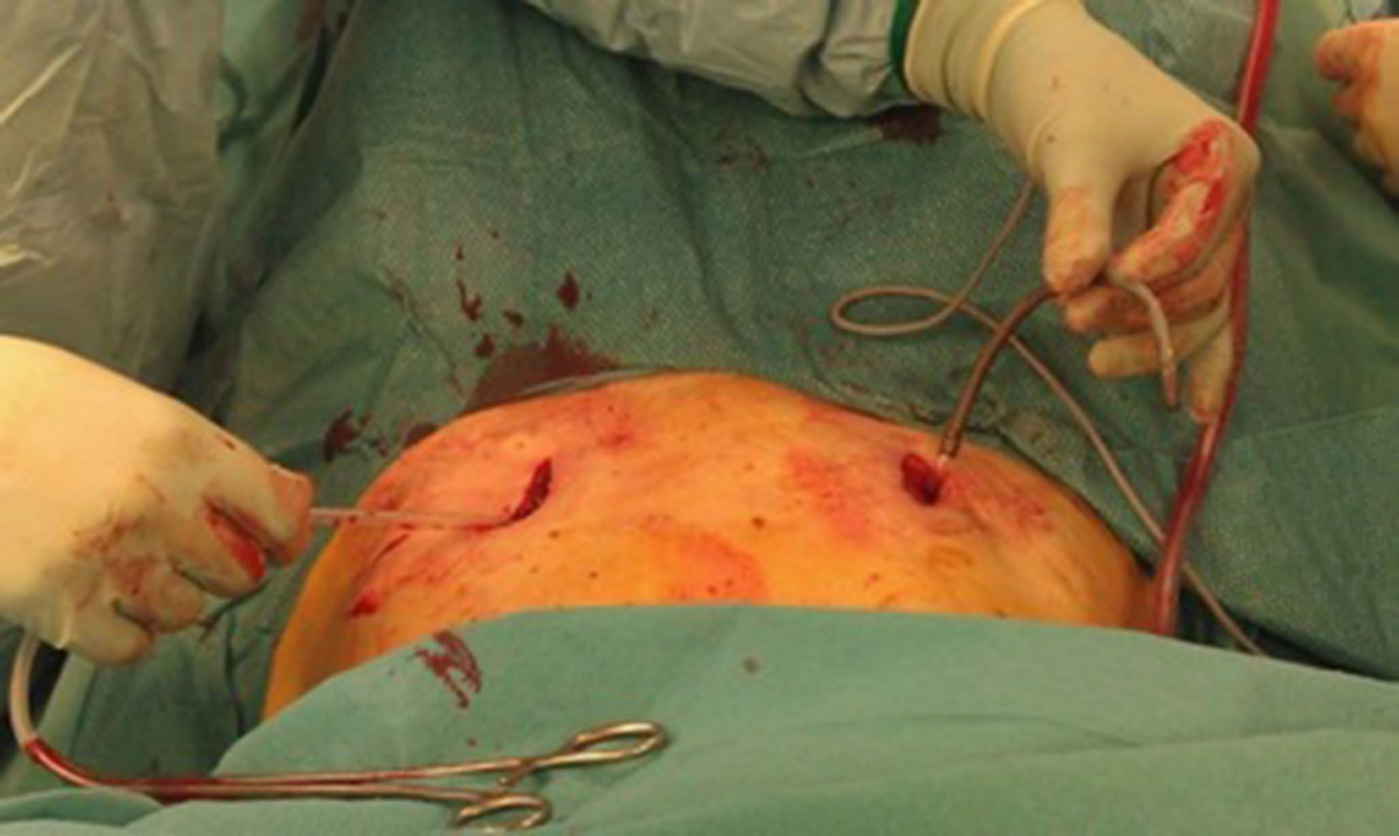
A curved titanium rod is fixed to one end of the drainage and then slightly pulled through the soft tissues to the contralateral side

**Fig. 8:**
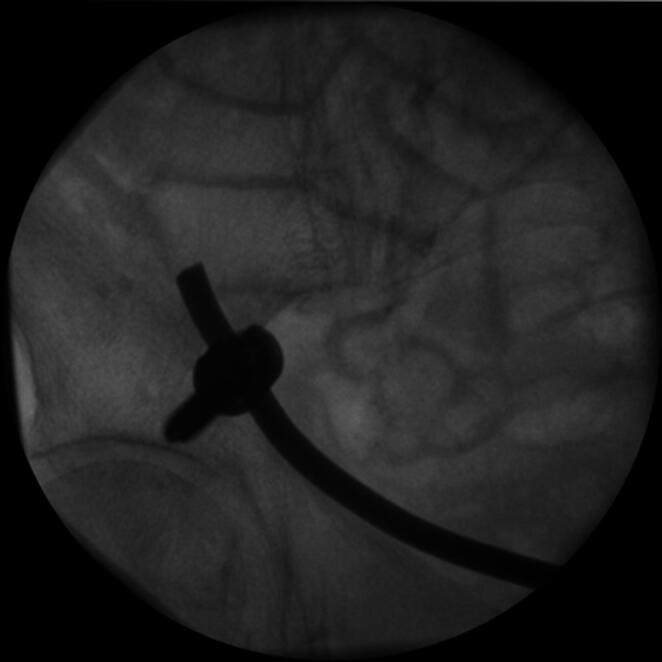
The titanium rod is now connected to the screw heads

**Fig. 9:**
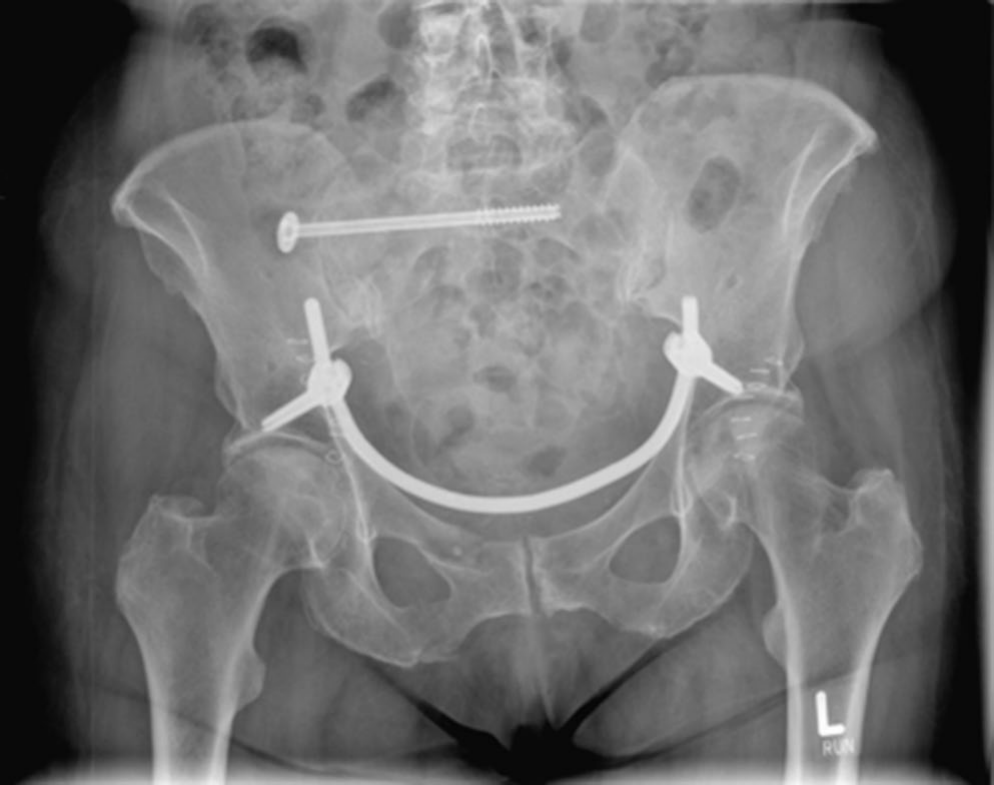
The titanium rod is finally locked after confirming the position of the rod as close as possible to both superior pubic rami by palpation

Next, to prepare the insertion of the titanium rod, a blunt preparation of the soft tissues is performed while the external iliac vessels are mobilized anteriorly. During this procedure, both superior pubic rami are used as landmarks for the preparation. Now a soft drainage is inserted on one side and tunneled through the soft tissues along the superior pubic rami posteriorly to the external iliac vessels until it reaches the contralateral incision (Figs. 5 and 6). A curved titanium rod of 6 mm diameter (Marquardt Medizintechnik GmbH, Spaichingen, Germany) is fixed to one end of the drainage and then slightly pulled through the soft tissues to the contralateral side (Fig. 7). The rod is now connected to the screw heads and finally locked after confirming the position of the rod as close as possible to both superior pubic rami by palpation (Figs. 8 and 9). Due to the locking of the rod in the screws, displaced fractures are reduced in a passive way.

The subcutaneous area and the skin are closed in relation to the different layers of soft tissues. Postoperative weight bearing is determined individually for each patient depending on the fracture morphology.

However before starting the mobilization all patients undergo a duplex ultrasonography of the vessels to exclude a thrombosis of the pelvic veins. A removal of the implants is not performed routinely, but only in the case of complications.

### Statistical analysis

Data were summarized with the use of descriptive statistics. Statistical analysis was performed using SPSS 27 (Statistical Package for the Social Sciences Version 27, IBM Corp., Armok, NY, USA). Medians and ranges were calculated.

## Results

Between 2012 and 2021, 34 patients were treated with an internal fixator, as described above, at a Level I trauma center in Germany. Decision making was based on our in-hospital treatment algorithm [16]. Either instability (FFP ≥ IIIa (*n* = 11), AO ≥ C1 (*n* = 4)) or severe pain in the anterior ring (*n* = 19) led to surgery.

### Patients’ characteristics

The median age of the population was 79 years (min.:65, max.:91, IQR:9). 26 patients were female (76.5%), while eight patients were male (23.5%). Most of the patients were categorized as ASA III (61.8%, *n* = 21), nine patients as ASA II (26.5%) and four patients as ASA IV (11.8%).

## Trauma history

Most injuries were a result of a low fall (64.7%, *n* = 22). Other causes were car accidents (11.8%, *n* = 4), falls greater than 3 m (5.9%, *n* = 2), run-over trauma (2.9%, *n* = 1), pseudarthrosis (2.9%, *n* = 1) and a pathologic fracture (2.9%, *n* = 1). Three patients could not remember a specific trauma (8.8%). Six patients (17.6%) were categorized as polytraumatized with an injury severity score (ISS) greater than 16.

## Fracture classification

Fractures resulting from simple falls (*n* = 22), pseudarthrosis (*n* = 1), pathological fractures (*n* = 1) and fractures where the patient did not remember any trauma (*n* = 3) were categorised according to the FFP classification [7]. Most of them were FFP IIb fractures (*n* = 10, 37.04%). Five patieets (18.52%) had a FFP IIIc fracture and four patients a FFP IIc fracture (14.81%). Three patients had a FFP IVa fracture (11.11%). Two patients had a FFP IVb fracture (7.41%) and one patient each sustained a FFP IIIa (3.7%), IIIb (3.7%) and IVc fracture (3.7%) (see Table 1).

**Table 1:** Baseline characteristics

Total *n* = 34	Total *n* = 34
Age [year (median, range, IQR)]	79 (65–91,9)
Gender [n %]	
female	26 (76.5)
male	8 (23.5)
Surgical time [min.]	75.31 min. ± 23.41
Fracture classification (FFP) [n %]* *n* = 27	
Ia	0 (0)
Ib	0 (0)
IIa	0 (0)
IIb	10 (37.04)
IIc	4 (14.81)
IIIa	1 (3.7)
IIIb	1 (3.7)
IIIc	5 (18.52)
IVa	3 (11.11)
IVb	2 (7.41)
IVc	1 (3.7)
Fracture classification (AO) [n %]** *n* = 7	
A1	0 (0)
A2	0 (0)
A3	0 (0)
B1	0 (0)
B2	3 (42.86)
B3	0 (0)
C1	2 (28.57)
C2	0 (0)
C3	2 (28.57)
ASA [n %]	
I	0 (0)
II	9 (26.5)
III	21 (61.8)
IV	4 (11.8)
Trauma mechanism [n %]	
ground-level fall (< 3 m)	22 (64.7)
car accident	4 (11.8)
fall > 3 m	2 (5.9)
run-over-trauma	1 (2.9)
pseudarthrosis	1 (2.9)
pathologic fracture	1 (2.9)
Polytrauma (ISS > 16) [n %]	
yes	6 (17.6)
no	28 (82.4)
Hospital stay [days(median, range, IQR)]	18.5 (8–54,15)

*Only fractures resulting from a simple fall were categorised using the FFP classification. Patients with high impact trauma, pseudarthrosis or pathologic fracture were excluded

**All fractures that were not the result of a simple fall were categorised according to the AO classification

Fractures which were caused by other reasons (*n* = 7) were categorized by AO classification [27]. Three patients (42.86%) had a B2 fracture, two patients a C1 fracture (28.57%) and two patients (28.57%) sustained a C3 fracture.

### Perioperative data

**Fig. 10:**
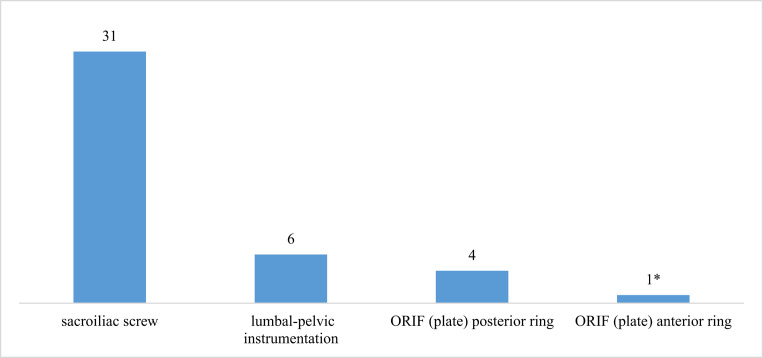
Additional stabilization procedures (several additional stabilization procedures per patient are possible). * One patient with anterior ORIF because of additional fractures of the acetabulum on both sides with bilateral hip dislocation. On one side, an anterior plate to fix the acetabulum was placed bridging the symphysis because fixation of the plate in the area of the fractured superior ramus of the ipsilateral pubic bone was impossible

The mean application time from skin incision to skin closure with sutures was 75.31 min (± 23.41). Except for one, all patients had an additional posterior ring fixation, most frequently minimally invasive stabilization with cement-augmented sacroiliac screws (91.18%, *n* = 31) (Fig. 10). The median time in hospital was 18.5 days (min.8, max.54, IQR:15).

### Intra-/postoperative complications

Complications during surgery occurred in four patients (11.76%). Three patients (8.82%) had a vascular injury (external iliac vein), which was repaired immediately by suture of the vessel. One patient (2.94%) suffered an injury of the bladder after local radiation of a gynecological tumor. This injury was fixed with a suture as well.

Postoperative complications occurred in six patients (17.64%). Four patients (11.76%) developed a seroma or hematoma including one infection, which led to revision surgery. One patient (2.94%) had a dislocation of the titanium rod because of an insufficient connection between screw and rod. Revision surgery was required 13 days after primary surgery. Thus, five patients underwent early revision surgery (14,71%). One patient developed necrosis of the bladder due to pressure of the curved rod, so removal of the implant was necessary (time to removal: 77 days). At this time the fracture was already healed. The allover revision rate was 17.64%. One patient (2.94%), who suffered a fracture as a result of a simple fall, died during hospitalisation from a non-surgical cause (cardiac arrest). Table 2 provides an overview of all surgery related complications.

**Table 2:** Complications *Several complications per patient are possible

	Total *n* = 34
Non-surgical complications* [n]	
Without non-surgical complications	14
Clavien-Dindo I	0
Clavien-Dindo II	14
Clavien-Dindo III	0
Clavien-Dindo IV	5
Clavien-Dindo V	1
Urinary tract infection	13
Pneumonia	3
Sepsis	1
Myocardial infarction	2
Thrombosis	6
Pulmonary artery embolism	1
Surgery-related complications* [n]	
Nerve damage	0
Bleeding	3
Infection	1
Implant failure	1
Hematoma/seroma	4
Urogenital complications	
-intraoperative	1
-postoperative	1

### Non-surgical complications classified by clavien and Dindo

Complications during the hospital stay were evaluated and classified using the Clavien-Dindo classification [28]. Fourteen patients (41.18%) developed a Clavien-Dindo type 2 complication. Type 4 complications occurred in five patients (14.71%), and a type 5 complication in one patient (2.94%). Table 2 provides an overview of all non-surgical complications.

### Mobility before trauma and after 12 month

Follow up data after 12 month were available for 11 patients.

Before admission to hospital, nine patients (69.2%) walked without any aids, and two patients (15.4%) used a walker for walking around. After 12 months, six patients (46,2%) did not need any aids for walking, and four patients (30.8%) needed a walker. Of all patients with available 12-month follow-up data (*n* = 11), the majority regained the same level of mobility as before the fracture (72.73%). For more information on mobility follow-up data and patient-reported outcome measures (PROMs), see Table 3.

**Table 3:** Follow up data: mobility, Barthel index, IADL (instrumental activities of daily living)

Mobility, Barthel index and IADL	[*n*, %, median, range; IQR]
Mobility before trauma (*n* = 13)	
-walking without aids	9 (69.2%)
-walking with walker outside	2 (15.4%)
-capable of walking only indoors	2 (15.4%)
Mobility after 12 month (*n* = 11)	
-walking without aids	6 (46.2%)
-walking with walker outside	4 (30.8%)
-capable of walking only indoors	1 (7.7%)
Barthel Index	
-before trauma (*n* = 13)	100 (60–100;15)
-after 12 month (*n* = 11)	85 (40–100;40)
IADL	
-before trauma (*n* = 13)	6 (3–8;2)
-after 12 month (*n* = 11)	3 (0–8;5)

## Discussion

As a result of demographic change, the number of fragility fractures of the pelvis is rising [29]. Yet there is still no consensus about the most appropriate stabilization technique in dislocated fractures of the anterior pelvic ring in geriatric patients.

The advantages of external fixation in dislocated pelvic ring fractures has been shown in several studies, but it is also criticized due to loosening, infections and especially in elderly patients its negative influence on an early mobilization [14, 18–20]. In contrast to this, minimally invasive procedures offer reduced blood loss, fewer soft tissue complications and infections, faster rehabilitation and a better pain control.

Due to this, the aim of the study was to describe a new technique of minimally invasive fixation of the anterior pelvic ring in geriatric patients and to analyze complications and outcomes.

34 geriatric patients were treated by using the submuscularly implanted internal fixator. With an median age of 79 years (min.:65, max.:91), a majority of female patients and multiple pre-existing illnesses (ASA III: 60.6%), the present study population showed the classical baseline characteristics of geriatrics. A ground-level fall as the main cause of fracture in geriatric patients is also well described in the literature [8, 30, 31].

The indications for this stabilization technique were a pelvic ring fracture with severe pain in the anterior ring, which leads to an increasing immobility, or an unstable fracture with a ventral dislocation. The decision regarding the most appropriate treatment was based on an in-house treatment algorithm [16]. According to the literature surgical stabilization of the posterior ring in stable fractures, especially FFP II, which are the most common pelvic fractures in the geriatric population, leads to improvement in pain and mobility after failure of conservative treatment [7, 14, 15]. However, in unstable fractures (FFP III and IV), the literature also recommends treatment of the anterior pelvic ring [15]. There is still no clear recommendation on when to address both the anterior and posterior ring, due to a lack of prospective studies. Keeping this in mind, severe pain in the anterior ring that led to immobilization, even in fractures that appear to be stable or with only minor dislocation, was a clear indication for us to stabilize the anterior ring with the internal fixator.

All but one patient received additional stabilization of the posterior ring, most commonly using percutaneous cemented sacroiliac screws (*n* = 31, 91.13%). Various studies have shown favorable outcomes in terms of pain relief [32, 33], functional results [34, 35], and quality of life [35] using this technique. Due to these findings and in combination with the advantages of minimally invasive procedures, the percutaneous sacroiliac screw fixation became the most frequent technique for a stabilization of the posterior ring [36].

Both intra-operative (11.76%) and post-operative (17.64%) complications were high in our population, the majority of these could either be addressed immediately intraoperatively or did not lead to permanent sequelae. Nevertheless the revision rates need to be discussed thoroughly. Kuttner et al. and Vaidha et al. first described a similar technique, utilizing a subcutaneously placed rod, which is fixed with two supraacetabular pedicle screws, known as ASPIF (anterior subcutaneous pelvic internal fixator) or INFIX (supraacetabular pedicle screw internal fixation device) [37, 38]. The indications for surgery were unstable B or C fractures. Kuttner et al. also included patients with severe soft tissue defects in the pelvic area, which was claimed to be a contraindication by Vaidha et al. Different retrospective case series, using the INFIX, have shown favorable short-term results regarding patients’ comfort, nursing care and rehabilitation [23, 37–39]. However, the most common complication was an irritation or neuropraxia of the lateral femoral cutaneous nerve (LFCN) in 8.3–57% of cases, wich was usually temporary [22–24]. Removal of the implant was performed routinely 3 to 6 months after primary surgery. 7% of all patients required early revision surgery because of loose of reduction or patients’ discomfort by placing the rod too deep. Other complications were surgical heterotopic ossification (36%) and late-onset infections (3.3%) [24]. There were no vascular injuries reported [24, 38]. In contrast to this, three patients of the present study had a vascular injury, specifically an injury to the external iliac vein, which could be repaired immediately. In our opinion, the main reason for this is the deep positioning of the implant, which requires a blunt dissection retroperitoneal close to the large pelvic vessels in contrast to the subcutaneous position of the INFIX. However, placing the rood directly on the bone provides both high biomechanical stability and protection of the vassels due to the submuscular position after implantation. With an early revision rate of 14.71% due to (hemato-)seromas (*n* = 4, including one infection) and one implant failure, this rate is twice as high compared to the INFIX. In contrast to the other publications, the present study investigated a geriatric population, in which most of the patients took anticoagulants (*n* = 17, 50%) and had relevant pre-existing illnesses (ASA III: 60.6%). This might be a reason for the higher number of hematoseroma compared to younger and healthier populations. Of the patients who developed a (haemato-)seroma in this study, three patients (75%) were taking anticoagulants and one patient was taking methotrexat, an immunosuppressive drug, which may have led to the infected seroma. A removal of the implant, however, was only necessary in one patient because of necrosis of the bladder and was not performed routinely, which the authors consider to be one of the major advantages of this procedure. A higher mortality due to surgical procedures in geriatric patients is well described in the literature [40]. According to this, the INFIX with its routinely removal was primarily used in polytraumatized, young patients (average age of 39–44 years, male > female patients). The INFIX was not primarily recommended for the stabilization of osteoporotic fractures. At 4.55%, the infection rate in the INFIX groups was also higher than in our population (*n* = 1, 2.94%). Furthermore, no nerve damage was observed in the patient cohort, in contrast to reports from other authors using the INFIX [22–24, 38].

Beyond that, Hack et al. investigated the stability of the submuscularly implanted internal fixator in comparison to the traditional external fixator [26]. Using adult human cadaver pelvis specimens, this biomechanical study has already shown the superiority of the internal fixator in terms of plastic deformation and stiffness, as well as equal results concerning the maximal load. Similar results showing the superiority of the INFIX compared to the external fixator were published by Vigdorchik et al. and Osterhoff et al., using synthetic pelvic bones [41, 42]. In contrast to the experimental study of Hack et al., characteristics such as the presence of osteoporosis are not taken into account in the studies by Vigdorchik et al. and Osterhoff et al.

The *anterior pelvic bridge* as another minimally invasive technique for fixation of unstable anterior pelvic ring fractures was first published by Cole et al. [43]. With the use of a subcutaneously placed internal fixation device, usually a precontoured low-profile reconstruction plate, a connection between an ipsilateral iliac crest and a contralateral pubic tubercle is built that bridges the fractured pelvic structures of the anterior ring. With this technique the author demonstrated positive clinical outcomes in terms of infection rates, implant failure and loss of reduction compared to a control group treated with an external fixator. With an infection in only one patient, this rate is comparable to the findings of this study (4% vs. 2.9%). Similar to the population of the present study, symptoms of a transient LFCN lesion were described in only one patient (*n* = 1, 4.2%). However, the implant was also removed routinely in this population. Furthermore, the study population of Cole et al. was much younger (mean age: 33y; range: 13-66y) compared to the geriatric patients of the present study. Whether this technique would also provide sufficient stability in fragility fractures with osteoporotic bones has not been proven.

Another minimally invasive technique for stabilizing anterior pelvic ring fractures was published by Rommens et al. 2020. They recommended using retrograde transpubic screws in pubic rami and more lateral fractures [25]. In contrast to our results, Rommens et al. had no vascular, neurological or urological complications in their retrospective analysis of 128 patients using retrograde transpubic screws. The reason for this could be a technically easier approach. However, this procedure is only recommended in minimally displaced fractures of the pubic rami [25]. In their population of both geriatric and non-geriatric patients, there were no differences in the rate of implant loosening or nonunion between the geriatric and the non-geriatric group. Implant-related complications on the other hand occurred more often in the geriatric group (10% vs. 5.9%). The allover revision rate, however, was higher in the population of this study (13% vs. 17.65%).

This study has several limitations. The first limitation is the retrospective study design, where incomplete data is a common problem. In addition, there is no control group, the sample size is relatively small, and selection or treatment bias could limit the information provided. In addition, not all patients had standardised follow-up examinations. Therefore it is difficult to draw a clear conclusion regarding the clinical outcome of this technique, as there is little information on follow-up and additional posterior ring stabilisation was performed. Nevertheless, to our knowledge this is the first study evaluating a minimally invasive technique for stabilizing dislocated anterior pelvic ring fractures in a geriatric population.

## Conclusion

The purpose of this present study was to analyze the short-term results of a new stabilization technique for anterior pelvic ring fractures in geriatric patients. The submuscularly implanted internal fixator offers many advantages, e.g. its minimally invasive technique, superiority in biomechanical stability, improvement in patient comfort, and no need for implant removal. Because of these advantages, the authors consider the submuscularly implanted internal fixator to be an alternative technique for stabilizing dislocated anterior pelvic ring fractures in a selected group of geriatric patients. However, because of its technical challenges this procedure should only be performed by experienced surgeons. As there is little information on follow-up, further prospective studies and long-term results are warranted to confirm our initial clinical impressions.

## Data Availability

No datasets were generated or analysed during the current study.
